# A Review of Pathway-Based Analysis Tools That Visualize Genetic Variants

**DOI:** 10.3389/fgene.2017.00174

**Published:** 2017-11-07

**Authors:** Elisa Cirillo, Laurence D. Parnell, Chris T. Evelo

**Affiliations:** ^1^Department of Bioinformatics – BiGCaT, Maastricht University, Maastricht, Netherlands; ^2^Jean Mayer-USDA Human Nutrition Research Center on Aging at Tufts University, Agricultural Research Service, USDA, Boston, MA, United States

**Keywords:** genome-wide association study, SNP, pathway analysis, epistasis, software comparison, data visualization

## Abstract

Pathway analysis is a powerful method for data analysis in genomics, most often applied to gene expression analysis. It is also promising for single-nucleotide polymorphism (SNP) data analysis, such as genome-wide association study data, because it allows the interpretation of variants with respect to the biological processes in which the affected genes and proteins are involved. Such analyses support an interactive evaluation of the possible effects of variations on function, regulation or interaction of gene products. Current pathway analysis software often does not support data visualization of variants in pathways as an alternate method to interpret genetic association results, and specific statistical methods for pathway analysis of SNP data are not combined with these visualization features. In this review, we first describe the visualization options of the tools that were identified by a literature review, in order to provide insight for improvements in this developing field. Tool evaluation was performed using a computational epistatic dataset of gene–gene interactions for obesity risk. Next, we report the necessity to include in these tools statistical methods designed for the pathway-based analysis with SNP data, expressly aiming to define features for more comprehensive pathway-based analysis tools. We conclude by recognizing that pathway analysis of genetic variations data requires a sophisticated combination of the most useful and informative visual aspects of the various tools evaluated.

## Introduction

### Pathway Analysis for Genome-Wide Association Study Data

Today, pathway analysis is routine with software or web services that accept and analyze different omics data, transcriptomics, proteomics with protein–protein interactions, and metabolomics. Methods and tools used to visualize and analyze these three main kinds of high-throughput data have been reviewed (Gehlenborg et al., 2010). Moreover, a decade ago genetic variation data, such as single-nucleotide polymorphism (SNP) originating from analyses of array-based genome-wide association studies (GWAS), began to be incorporated into pathway analysis (Wang et al., 2007). Since then, the method was applied to other types of studies involving SNPs such as: epigenome-wide association study (EWAS) (Shimada-Sugimoto et al., 2017) or sequencing-based GWAS (Guodong and Degui, 2013). Although genetic association research is advancing rapidly, and especially GWAS studies are commonly performed for the genotype–phenotype investigation, biological interpretation of those data remains a challenge; especially when interpretation concerns connecting genetic findings with known biological processes (Manolio, 2013). Application of pathway analysis to SNP data is a valid approach to meet this challenge for different reasons: first, because of the polygenic nature of complex diseases, such an approach holds the promise to contextualize the SNP data better and to suggest novel interpretations of the results based on prior knowledge of genes and pathways (Wang et al., 2010). Second, a typical display of genetic association results consists of the few SNPs showing strong evidence for disease or phenotype association (generally *p*-value <1e^-8^), but it is also well-known that these few associated SNPs often have only a modest effect on disease risk (Zhong et al., 2010). Thus, examining the cumulative effects of numerous variants and visualize them at the pathway level, can empower detection of genetic risk factors for complex diseases (Manolio, 2013; Mooney and Wilmot, 2015).

We believe that data visualization, in the form of interactive pathway diagrams and/or gene–gene biological interactions such as genetic networks, enhances interpretation of scientific data, understanding the conclusions drawn, and discussing follow-up research questions (Villaveces et al., 2015). Currently, programs like Gene Set Enrichment Analysis (GSEA; Mootha et al., 2003; Subramanian et al., 2005), DAVID (Huang et al., 2009a,b) or g:Profiler (Reimand et al., 2016) display the pathway analysis output with lists, plots, or link to the pathway diagrams. However, we believe that providing an interactive pathway diagram or network visualizations with metadata from other sources, aids in understanding the question, problem, or relationships among the data entities. Thus, interpretation of SNP data would benefit from pathway-based approaches accepting of genetic variation, so that allele-specific relationships are displayed.

Recently, several step by step guides (Wang et al., 2010; García-Campos et al., 2015; Mooney and Wilmot, 2015; Kao et al., 2017) were published as reviews, describing and providing recommendations on how to use different pathway analysis methodologies, which are especially applicable to GWAS data. The main features to consider are: (i) make certain that GWAS analysis is performed according to standard guidelines; (ii) choose curated and up-to-date pathway collections; (iii) filter the list of gene sets to avoid bias related to size, a common limit is between 10 and 200 genes, and map the SNPs to genes based on location or linkage disequilibrium (LD); (iv) choose the method according to the statistical hypothesis to be tested; (iv) report the results and if applicable visualize them in order to improve comprehension. Regarding the point of the statistics, Wang et al. (2007) were among the first to publish a pathway-based GWAS analysis using a statistical method adapted for genetic variation data. The authors modified a GSEA algorithm, initially designed for pathway analysis of gene expression data (Subramanian et al., 2005). Since the adaptation of GSEA by Wang et al. (2007), researchers have developed other statistical methods for pathway-focused analysis of associating SNPs. Currently, existing methodologies for the analysis of GWAS gene sets are based on over-representation analysis, enrichment analysis, functional class score, and pathway-topology (García-Campos et al., 2015; Mooney and Wilmot, 2015; Kao et al., 2017). The recommendation is to apply multiple methods to capture different genetic effects and identify robust gene set associations (Mooney and Wilmot, 2015). However, only a few of these new algorithms were implemented in user-friendly tools, possibly because pathway-based approaches still have many technical challenges to overcome (Wang et al., 2010). Beside the main focus of data visualization, the literature search performed in this review, allow also to verify if the existing pathway analysis algorithms for genetic variations are available in user-friendly pathway tools that provide visualization options. In general, it is recognized that improving and standardizing the practice of this methodology, not only will improve the comparability of the results of gene set analysis, but also will allow a better evaluation of related polymorphisms both in the same and in different but functionally related genes. This step potentially would increase the power to detect causal pathways and disease mechanisms, using SNPs with significant associations and those in LD with functional variants. Moreover, it can point toward integration of omics data, where the additional molecular information could verify or predict the functional effects of the associating SNP (Wang et al., 2010).

We identified a major shortcoming concerning pathway analysis programs for SNP data: genetic variation analysis have not been combined commonly in user-friendly pathway analysis tools, that provide both interactive visualization options, enabling the exploration of the data and metadata on the pathway diagrams, and existing statistical methods specifically designed for SNP analysis. For example, one allele of a pathway entity might allow the bioprocess to continue while a second allele curtails pathway flux. Then, visualizing on a pathway map the effect of variants associated with elevated risk of disease, can indicate biological and biochemical insufficiencies (and/or vulnerabilities), which then can be made more informative if placed within depictions of the affected cell or organ, or other data related to the entities of the pathway. For instance, the rs11591147 SNP which maps to exon 12 of *PCSK9* gene, directing an amino acid change Glu670Gly is a proper example to understand the potential of dynamic pathway visualization. This variant encodes a gain-of-function allele in *PCSK9* that influences inter-individual variation in low-density lipoprotein (LDL) cholesterol levels between African-Americans and European-Americans (Ding and Kullo, 2008). In the WikiPathways database (Kutmon et al., 2016) there is the proprotein convertase subtilisin/kexin type 9 (PCSK9) mediated LDL receptor degradation pathway (WikiPathways ID: WP 2846) that represents the key role of PCSK9 in the regulation of the LDL-cholesterol level. This pathway can be dynamically explored with the PathVisio tool (Kutmon et al., 2015), in which not only the different entities of the pathway will show extra information through their hyperlinks with various sources (e.g., gene, protein, disease databases, etc.), but also genetic variation data with hyperlinks to SNP databases, gene expression data, and interaction values can be displayed on the pathway diagram. This multiple data visualization combines different types of information that allow the researcher to describe more easily the possible effect(s) of the genetic variant in the bioprocess with the additional support of other data. Even if genetic variation data are not available, the *in silico* prediction variant score such as SIFT (Kumar et al., 2009), Polyphen (Adzhubei et al., 2010), or CADD (Kircher et al., 2014) can be used in the pathway diagram to envision the possible consequences of the variant on gene interactions. Hence, this type of interactive pathway visualization is important in facilitating use of the data for particular instances.

Lastly, this type of pathway analysis visualization is applicable for SNPs data originating from different types of studies (e.g., EWAS and sequencing data), but it can also support the interpretation of specific phenomena such as epistasis or gene–gene interaction. Epistasis is yet another manner in which connections within a pathway are different in different individuals, where two alleles mapping to different loci associate in concert with a phenotype, but where those two alleles individually show no phenotype association (Wei et al., 2014; De et al., 2015). Consider, for example, that pathway endpoints are a phenotype, clinical indicator of health or disease status, or disease itself. Then, the epistatic relationships can be indicated by epistatic- or “e-edges” that serve to connect distinct pathways or different nodes within a single pathway in this conditional relationship. The pathways linked by such “e-edges” would give support to co-function and/or co-regulation with regard to the given phenotype of interest. In addition, the nodes within the GWAS-identified pathways, i.e., the main effect associations, can be used to focus the genetic landscape in the search for epistatic relationships as opposed to searching for epistasis across the entire genome.

However, genetic variants currently cannot be combined easily in pathway representations because it is not clear how to visualize and interpret variation data once connected programmatically to pathway content. In this review, we sought to investigate how to dynamically visualize genetic variations in a pathway context using user-friendly tool. First, we performed a systematic review of articles that analyzed genetic variants using pathway based methods in order to identify and describe the visualization options of the tools resulting from this literature review. The purpose of the tool evaluation relates directly to the need to combine SNP data, such as those from GWAS results, with biological context in order to better understand results in a disease context. Second, we performed a use case with the tools identified, testing a computationally derived epistatic dataset of gene–gene interactions for 12 candidate genes in obesity risk, in order to evaluate how genetic variant analysis of epistasis is tackled by the tools. Taking a visualization point of view, we report the features and the potential of the different software. Reviewing the articles, we also collected current statistical methodologies that have been applied in pathway-based analysis of GWAS data, and we report those without discussing in detail.

## Materials and Methods

This review follows criteria developed by the PRISMA statement (Moher et al., 2009).

### Search Strategy

In order to assemble an overview of visualization approaches used in studies that applied pathway-based analysis to genetic association studies fully reflecting current practices, a keyword search for “Pathway Analysis” in PubMed and Medline (July 2014) was conducted. The literature research was performed using EndNote X7. The search yielded 2,231 articles from January 2005 through August 2014, 2,184 remained after removing duplicates, 15 others were added based on suggestions by experts in the field. Subsequently, these articles were screened manually by reading title and abstract. We retained only those 264 articles describing pathway-based analysis with genetic variation, and these articles were studied in detail. Retaining the 65 most relevant papers, all from 2007 through 2014, we aggregated the results with key features of the analysis, summarized in Supplementary Table 1. In order to update the manuscript with additional visualization tool for GWAS pathway analysis, we performed a second PubMed search in January 2017 using the keyword “Pathway Analysis” for title and abstract, and date of publication from August 2014 to present. We obtained 2,774 articles that were scanned by title. Several articles describing GWAS pathway analysis tools were found (see Supplementary Table 2), but only one PathVisio (Kutmon et al., 2015) presented interactive visualization features in pathway diagrams. This one was included and described in the tool paragraph, and reported in **Table 1** together with the other four tools previously identified. Details of the 65 relevant articles selected with the literature search are given in Supplementary Table 1. Columns describe specific features extracted from each study: type of data and variants, algorithm used, and bioinformatics tools used with visualizations. Because we did not select the articles based on the type of variants utilized, but on the type of analysis performed (keyword used: “Pathway Analysis”), we also identified articles where the variants participating in the genotype–phenotype association originated from sources other than SNP arrays. In the 65 articles: 57 were based only on GWAS data, four on GWAS plus expression data, one on GWAS plus epigenetic data, two used known somatic mutations, and one using next generation sequencing data. In all studies, the resulting SNPs were investigated using pathway-based analysis, and only three studies also analyzed copy number variants and/or indels (Ghosh et al., 2013; Leiserson et al., 2013; Lee et al., 2014).

**Table 1:** Summary of the main features of the pathway-based analysis tools evaluated.

Features	Caleydo	IPA^TM^	MetaCore^TM^	PathVisio	Path
Availability	Free download	Private	Private	Free download	Free download
Type of genetic variants data	CNVs	SNPs	SNPs	SNPs	SNPs
Variants data format	.csv, .txt, .gct	.xsl, xslx, .txt	VCF	.csv, .txt	LINKAGE pre-mapped, QTDT
Pathway collections and size	KEGG with 518 pathways, WikiPathways with 743 curated *Homo sapiens* pathways	Private collection with 662 curated pathways	Private collection with 1,662 curated pathways	WikiPathways with 743 curated *Homo sapiens* pathways	KEGG with 518 pathways
Applications for pathway-analysis	enRoute, Entourage	Enrichment Analysis	Enrichment Analysis Workflow	Enrichment Analysis	UNPHASED
Gene description	Present	Present	Present	Link to the gene database	Present
Variants data visualized on pathway	YES	YES	YES	YES	Not known because of the bug
Variants description	Not present	Not present	Not present	Links to the variants database	Not known because of the bug
Linkage disequilibrium map	Not present	Not present	Not present	Not present	Present
Presence of other omics data	YES	YES	YES	YES	NO
Version of the tool	V.3	V.01-08	V.6.29	V.3	V.1
URL	www.caleydo.org/	www.qiagen.com/ingenuity	https://portal.genego.com/	www.pathvisio.org	http://genapha.icapture.ubc.ca/PathTutorial/

*CNV, copy number variation; SNPs, single-nucleotide polymorphisms; VCF, Variant Call Format.*

### Overview of Pathway Analysis Tools for Genetic Variation Data

Although some algorithms are available as web services or installable software, no generally accepted implementation for the visualization of SNP results exists. From the literature search, we found the following bioinformatics tools able to visualize the significant variants in a pathway: IPA^TM^ of QIAGEN’s Ingenuity Pathway Analysis (2016; QIAGEN Redwood City^1^) (Inada et al., 2008; Helleman et al., 2010; Ngwa et al., 2011), MetaCore^TM^ from Thomson Reuters^2^ (Song and Lee, 2013), Path^3^ (Daley et al., 2009; Zamar et al., 2009), and Pathvisio 3 (Kutmon et al., 2015). In general, very few tools support pathway visualization of genetic variants. In addition, the Gehlenborg et al. (2010) review mentions a visualization tool not found in the articles reviewed. This tool is called Caleydo^4^ and it depicts only copy number variations (CNVs). We describe in the Section “Results,” the five tools mentioned here with a specific focus on the visualization options for the genetic variants. However, some relevant command line tools were also detected in the literature search, but we do not describe these because of the absence of user-friendly visualization features.

We also evaluate three of the five tools selected from the literature search, using an available epistatic dataset (De et al., 2015). Because the tools do not only require different formats, but also have different features, we could not use this dataset for Caleydo and Path. For these tools the evaluation of the visualization was assessed using the default dataset provided by the software and the tutorials.

### Dataset of Epistatic Interaction

An epistatic dataset from De et al. (2015) is chosen to evaluate the SNP visualization in the biological pathways of three tools retrieved from a literature search: IPA, MetaCore, and PathVisio. The dataset consists of a list of SNPs with significant epistasis interactions (SNP–SNP connections) calculated from a gene–gene interaction epistasis network of 12 candidate genes for obesity risk *(BDNF, ETV5, FAIM2, FTO, GNPDA2, KTCD15, MC4R, MTCH2, NEGR1, SEC16B, SH2B1, TMEM18)*. SNPs were extracted from the 12 genes following specific criteria: 500 kb window of the gene, exclusion of SNP with minor frequency allele <0.05, exclusion of SNP that shows LD of *r*^*2*^ > 0.8, and imputation of missing genotypes. The resulting SNP dataset in the study was 1,191 SNPs with genotype data available for 1,141 obese individuals (body mass index >30 kg/m^2^). Genotyping was performed with Affymetrix 500 K mapping array and the Affymetrix 50 K supplemental array. A statistical epistasis network (SEN) (Hu et al., 2011) was utilized to characterize the interactions between genetic variants from the 12 obesity genes, resulting in a list of 58 SNPs with significant mutual information. This value corresponds to a weight of each SNP and each pair of SNPs in SEN. In addition, it quantifies the strength of the interaction outside of the individual main effects of a SNP pair on the phenotype. We used the 58 SNPs as input to the three tools selected for the visualization evaluation. Describing the advantages and disadvantages of the tool features, we try to understand which tool can facilitate the interpretation of the SNPs in the pathway context.

## Results

### Pathway-Based Analysis Tools with Visualization Options

The evaluation of five pathway-based analysis tools—Caleydo, IPA, MetaCore, Path, and PathVisio—that support incorporation of genetic association data demonstrates: first, how polymorphism data can be visualized and analyzed in a pathway-based environment, and second, how different information and experimental data can be combined for analysis and visualization. Pathway content provides the biological processes in which GWAS-identified genes are known to be involved and shows other genes related by common function that may not pass GWAS significance thresholds. Integration of other types of genomics data as accepted by these tools, often in combination with bioinformatic pipelines for data processing, permit evaluation of different transcriptomics outcomes in subjects with a specific genotype or phenotype, and some tools allow also integration of metabolomics results.

The five tools are designed to visualize the data on different pathway collections originating from different databases. Path refers to KEGG^5^ (Kanehisa et al., 2012), PathVisio to WikiPathways^6^ (Kutmon et al., 2016), and Reactome (Fabregat et al., 2016), Caleydo to both KEGG and WikiPathways; while MetaCore and IPA use their respective curated pathway collections.

### Tool-Specific Visualization Details

MetaCore is a software suite suitable for functional analysis of different omics data, including expression data and genetic variation data. One of MetaCore’s relevant applications for pathway analysis is the Enrichment Analysis Workflow, which calculates enrichment *p*-values in different types of gene sets within the uploaded dataset. These gene sets originate from curated pathways, networks of related genes derived primarily from literature evaluation and from the Gene Ontology lexicon. We performed an example analysis using the 58 SNPs with significant epistasis interactions as input. As the tool accepts variants in a Variant Call Format (VCF) file, we formatted the input data accordingly. The results of this analysis recognized 13 objects, limited to just one SNP per gene. Different outputs such as pathway maps, gene ontology (GO) processes, process networks, and diseases (symbolized by biomarkers) are listed as part of the result (Supplementary Figure 1). All list items are clickable, allowing more detailed visualization of the different items. The resulting pathway maps are ordered by enrichment *p*-value, with false discovery rate (FDR) corrections. The FDR calculation considers the *p*-value of each network map and its rank given the total number of maps in the entire set of pathway maps. The list also contains the ratio of significant genes in the dataset over the number of genes in the pathway. If one pathway in the list is selected, a pathway map is displayed. In our example, the first pathway of the list is “retinal ganglion cell damage in glaucoma” in which two genes appear to be colored bright and illustrated that they present the input SNPs with a red colored bar (Supplementary Figure 2). Clicking a gene symbol displays detailed information about the description of the gene and encoded protein for human, mouse, and rat. Clicking the red bar yields details for the uploaded data of that gene, in this case the SNP rs ID. In the example pathway, two genes show data: *BDNF* with rs10835210 and *BnaC2 (ASIC1)* with rs1108923. It is remarkable to notice that *ASIC1* is not in the list of the 12 obesity genes of the study selected. Indeed, the SNPs from the obesity-epistasis dataset (De et al., 2015) were extracted taking into account a window of 500 kb from the obesity genes, but MetaCore assigned SNPs only positioned within a gene region. This is also the reason why the total SNPs identified by the analysis is 13 and not 12. In this case, rs1108923 is selected in the dataset because it maps to the upstream region of the obesity gene *FAIM2*, but the tool considers this variant to be within the region of *ASIC1*.

QIAGEN’s Ingenuity Pathway Analysis, IPA is a web-based application for data analysis in pathway context. Although the IPA environment is amenable to different types of analysis (i.e., Metabolomics, microRNA, Toxicology, etc.), our objective is to highlight aspects of pathway analysis. After uploading the list of 58 SNPs with the significant epistasis interactions value, the program automatically displays an overview page with information such as the number of SNPs recognized by the tool, in this case 22 SNPs of 58 were mapped. In addition, a table is shown with Entrez gene IDs and affiliated information such as cellular location, type of gene, and interacting drug. Clicking on one of the gene names listed, it displays a link to a description gene page for human, mouse and rat, in which additional information about the gene functionality are provided. In this overview page, there is a possibility to perform different analysis as was mentioned above. We opted to the Core Analysis that includes the enrichment pathway analysis. However, such analysis takes into account the genes in which the 22 SNPs were mapped and not the SNPs themselves. The result page, as in MetaCore, lists several output such as: canonical pathways, diseases and function, regulators, and networks. The canonical pathway visualization is a list of enriched pathways ranked by *p*-value and percentage of the overlapping genes mapped against the total number of those in that pathway. Selecting a pathway prompts IPA to offer several views that depict different items within the top significant pathways such as bar charts, and stacked bar charts. The pathway visualization is displayed under the network tab, where genes with different colors and shapes are shown as clickable nodes that link with additional information related to that gene, including biochemical elements, metabolites, and references curated by IPA team (Supplementary Figure 3). At this level, further information about SNPs related to the genes is not visualized and reported.

PathVisio 3 is a pathway editor, visualization, and analysis software. PathVisio core features related to visualization are listed in a main panel where pathway diagrams can be drawn, and the entities of the pathway can be displayed in different ways according to advance data visualization options. There is a side panel called backpage where data and other visualization features are shown. Some of these features are related to the advanced options provided by plugins. Developed by any user, these plugins are extensions of the PathVisio system that do not change its core functionalities. Two of these plugins, BiomartConnect^7^ and RegInt plugin^8^, add functionalities related to genetic variants. BiomartConnect enables visualization of biological information in the backpage, retrieved with the Ensembl BioMart tool^9^, with which variants also are accessible. With this plugin the variants, stored in the Ensembl database and located in any gene selected from a pathway diagram, are visualized in the backpage. Moreover, additional SNP information like SIFT and Polyphen predictive scores is available and possible to display in the backpage. The RegInt plugin enables one to upload and visualize user data on the pathway, in the form of an interaction file. This file contained a data column listing the 58 SNPs and another listing the genes in which those SNPs are located. For the detailed input format check plugin instructions in Github^10^. We used the RegInt plugin to display the 58 epistatic SNPs. First, in the main panel, we opened a pathway diagram presenting at least one of the genes related to the 58 SNPs from the WikiPathways collection (see text footnote 6), a pathway database linked to the software. Then, in the backpage the SNPs related to the gene selected in the pathway are displayed. The number of SNPs visualized depends on the data uploaded. In our case we selected from WikiPathways the “brain-derived neurotrophic factor signaling pathway” (WP2380), that presents two *(BDNF* and *SH2B1*) of the 12 genes of the epistatic dataset (Supplementary Figure 4). From a biological prospective this type of visualization allows two types of investigation: one at the gene level where the relation between genes with significant epistatic SNPs can be explored in the pathway. The other one at the SNPs level, where the list of the epistatic SNPs is shown in the backpage and their effects can be explored further. Moreover, a SNP hyperlink that connects to a variant database in which the SNP description is provided, is a useful feature to speed the research into SNP function.

Caleydo is an open source software with three applications for data visualization: StratomeX (Lex et al., 2012), enRoute (Lex et al., 2013), and Entourage (Partl et al., 2013). StratomeX organizes different data from cancer patients, and retrieves disease information from TCGA datasets^11^. Packages that are of interest for pathway analysis are the Entourage view, which investigates interdependencies between pathways, and the enRoute view, which analyses experimental data in pathway context. The Entourage view compares pathway maps selected from the same or different pathway collections. A notable aspect is the visualization of pathway interconnectivity between selected pathways for specific genes (Supplementary Figure 5). This useful feature enables deeper insight because it depicts how a gene observed in one pathway might have different roles in an interconnected process. These interconnections are intuitively displayed with colored lines that connect the selected gene from the main pathway to its occurrence in other pathways. Lastly, enRoute allows selection of a subset of genes in a pathway, and these selected genes can be associated with experimental data from TCGA in which CNVs also are shown. Caleydo provides this type of visualization and analysis only for a specific set of experimental data (i.e., TCGA dataset), and for this reason it was not possible to upload the list of 58 epistatic obesity SNPs for the use case.

**FIGURE 1:**
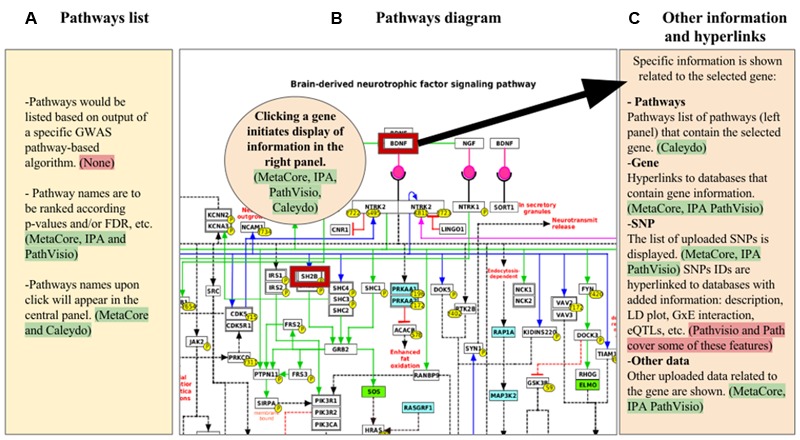
Mock-up visualization of the combination of useful features to apply for GWAS visualization and analysis in pathway-based tools. The panels show: **(A)** list of pathways obtained from a specific GWAS pathway analysis algorithm; **(B)** pathway diagram selected from one of the pathways listed in the panel **(A)**, where genes with GWAS hits are highlighted (red border); **(C)** other information with hyperlinks related to several types of data with regard to the gene selected from the panel **(B)**, that could be displayed in expandable/collapsible lists. Highlighted green are the tools in which the specific feature described is present, red highlights indicate features that are either not present or partially present in the tools reviewed.

Path is specifically designed for GWAS analysis, connects GWAS results with information retrieved from nine common bioinformatics resources (NCBI, OMIM, KEGG, UCSC Genome Browser, Seattle SNPs, PharmGKB, Genetic Association Database, dbSNP, The Innate Immune Database), and supports visualization of the integrated data. Path uses UNPHASED (Dudbridge, 2006) for statistical analysis and retrieving information on SNP–SNP associations from the different bioinformatics resources. The only pathway resource included is KEGG. Visualizations mainly consist of charts, plots, and summary tables that list genes, SNPs, SNP associations, and gene–gene interactions. Importantly, Path is specifically directed toward GWAS studies, showing specific association results, and lists of genes, SNPs and LD plots. The pathway visualization using KEGG data shows genes with significant SNPs highlighted in red similar to those shown in **Figure 1**. Currently, this type of visualization is not available because Path does not work properly due to unfixed bugs, which the authors have decided not to address at the moment. For this reason, it was not possible to perform the use case with the epistatic obesity SNPs. However, Path-2 is released^12^, in which the authors provided the PLINK-based single-SNP association analyses (logistic/linear regressions, family-based analyses) and Pathway/Ontology association analyses [SNP Ratio Tests SNP Permutation Tests, Nyholt Pathway Tests, Sidak Pathway Tests, Association LIst Go (gene ontology), AnnoTatOR (ALIGATOR) Tests]. This version of the tool is still relevant for specific pathway analysis with GWAS data, but the type of visualization provided no longer presents an interactive pathway diagram in which data are shown.

### Statistical Methods in Pathway Analysis Tools

The variants from the 65 articles retrieved by literature search, were evaluated for pathway assignment using different algorithms that were not always well described. When they were, the authors always provided the *p*-value of the variant from the genotype–phenotype association (Yu et al., 2009). The different algorithms used in the pathway-based methods aggregated SNP or gene scores to assign a *p*-value to a pathway. The association of a SNP to a particular gene is normally evaluated using a cutoff for SNP significance in a specific gene neighborhood region. Then, *p*-values assigned to each pathway can be calibrated and adjusted for some biological event such as LD patterns and co-location of functionally related genes. Such biological events can be evaluated differently by different algorithms, which can affect the results and suggest other conclusions. Researchers have developed different statistical methods for analysis of associating SNPs (Supplementary Table 1). Approaches include LD calibration and identification of associated pathways (Panagiotou et al., 2012), and comparison of different algorithms, which revealed advantages and disadvantages of the statistics used for a specific GWAS dataset (Gui et al., 2011; Evangelou et al., 2012; Fehringer et al., 2012; Jia et al., 2012). These articles compare different statistical methods tested in GWAS datasets, evaluating the lists of enriched pathways. Although not all algorithms listed in Supplementary Table 1 have been compared, we reported the conclusive judgment of the comparison performed in certain studies. Some of the most sensible statistical methods include the adaptive rank truncated product (ARTP) (Evangelou et al., 2012), the modified summary statistic (mSUMSTAT) (Panagiotou et al., 2012), and the raw data-based algorithms implemented in PLINK (PLINK set-based test) (Gui et al., 2011; Jia et al., 2012). These algorithms were shown to be the most powerful for detecting genes that could be used further by pathway analysis tools (Gui et al., 2011; Evangelou et al., 2012; Panagiotou et al., 2012). It is difficult to make a single and objective preference of one specific method because results of pathway-based analysis for GWAS data vary by method. Even the overlap of shared pathways can be quite limited because each algorithm has its own evaluation focus on disease associations (Jia et al., 2012), and some examples concern different calculations of values, including pathway *p*-values in ARTP, or the mean value of a gene with the significant SNP in mSUMSTAT.

From the tools analyzed, MetaCore, IPA, and PathVisio present a statistical analysis of the data provided. Instead Caleydo and Path provide only data visualization on pathway graph and not statistical methods for pathway analysis. MetaCore, PathVisio, and IPA perform pathway analysis in an automated fashion. The first tool uses an over-representation method on the gene list annotated from the variants present in the VCF provided as input. MetaCore employs a hyper-geometric model to determine the significance of the enrichments. PathVisio also uses an over-representation analysis and it is based on methods adopted in the MAPPFinder tool (Doniger et al., 2003) with settings designed for gene expression data. Finally, IPA utilizes a method for combining *p*-values. In the over-representation test, an association for each gene in the dataset is first calculated, then a threshold is used to determine which genes are significantly associated. The proportion of significantly associated genes within a target pathway is compared to the proportion of significantly associated genes among all genes outside the target pathway (Mooney and Wilmot, 2015). In the method applied in IPA, a *p*-value associated with a pathway is calculated using the right-tailed Fisher’s exact test that is equivalent to the hypergeometric test (Rivals et al., 2007). This *p*-value measures the likelihood that the association between a set of genes with a significant SNP identified by GWAS and a pathway arose by chance. In this method, the *p*-value for a given process annotation is calculated by considering (i) the number of genes with a significant SNP that participate in that process and (ii) the total number of genes that are known to be assigned to that process in the selected reference set. Further details on how IPA identifies pathways reaching significance were not provided (IPA webpage, June 23, 2016, date last access).

## Discussion

### Overview of the Comparison: Benefits and Limitations of the Tools

Comparing the five tools described above makes evident that each uses different interactive ways to combine experimental data with information about genes, metabolites, and pathway relationships (**Table 1**). A mock visualization of the beneficial and applicable features observed in the different tools (green highlight), and the new characteristics that enhance the visualization and analysis of SNP data in pathway-based analysis tools (red highlight) is shown in **Figure 1**. The five investigated tools share some similar and effective visualization approaches, such as depicting significant pathways that contain genes in the analyzed data by list view. These lists are generally ranked by enrichment ratios, *p*-values or FDR scores. Another common and useful strategy is to highlight genes for which pathway data are uploaded by the user, with an option to uncover gene details via hyperlinks.

A general problem in pathway-based visualizations is the efficient display of information about genes that appear in multiple pathways and thereby interconnect those pathways. Caleydo offers an attractive solution in allowing interactive and automatic visualization of subpathways of genes present in other pathways. Caleydo uses this subpathway approach to indicate when the dataset has information about genes in a given pathway. This demonstrates how experimental data can be combined with different types of knowledge about gene relationships and permits an increased understanding of experimental results that might act in concert. Caleydo provides this type of visualization and analysis only for a specific set of experimental data (i.e., TCGA dataset). It would be a large improvement if this same approach were used to automatically select the relevant genes in the pathways based on the GWAS statistical parameters such as SNP *p*-value or effect size beta, which in turn could offer an assessment of allele effects on pathway output, or other omics datasets.

A strength of PathVisio, on the other hand, is its enabling of this feature to permit visualization of variants in pathways sourced either from a public repository like Ensembl or from user data. However, PathVisio lacks the interactive visualization that links entities of different pathways, as it described in Caleydo. In this context, MetaCore depicts related experimental effects of genes known to be connected via membership in a pathway, protein–protein interactions, co-citation, or co-expression in other experimental datasets with network visualization. MetaCore’s network settings can be used to view or hide specific interaction mechanisms, such as binding, influence on expression, phosphorylation, or cleavage. IPA’s approach is similar to that of MetaCore. After running the enrichment analysis, IPA lists the most represented processes, such as canonical pathways, networks, upstream regulators, diseases, and biological functions. In this way, the user subjectively decides which information to use and how to integrate it. Finally, Path has some methods to integrate GWAS data in pathway analysis. Path’s basic data visualization of pathways uses the common strategies described above, and data integration focuses specifically on genetic information and on gene–gene interactions. Path’s representation also includes an LD plot, useful and important support for GWAS interpretation.

### Suggested Improvements for Data Integration in Pathway-Based Analysis Tools

As early as 2005, the importance of effective approaches to visualization was noted through interviews and observations of current work practices (Saraiya et al., 2005). That report highlighted different aspects of pathway visualization, and suggested future developments to improve the researcher’s job. Our comparisons indicate that most of those recommendations have been implemented. Two examples are the options to automatically search for relevant pathways containing genes from an uploaded dataset, and access to periodically updated pathway libraries. We have presented different types of visual strategies used in currently available tools that, for a specific gene set, support the connection with various kinds of pathway information including significant pathways, metabolites involved therein, and related diseases. With many different types of high-throughput data now readily available, including gene expression, metabolomics and protein–protein interactions, methods for integrated analysis and visualization are greatly needed (Gomez-Cabrero et al., 2014). Visual strategies are particularly important for data from high-throughput experiments that provide information about many genes, facilitating evaluation of potential interactions between affected genes. This potentially can speed the investigation of the SNP effect in the pathway. Indeed, highlighting the relevant items related to the research question can reduce the process of investigating pathways singly. Moreover, alternative visualizations such as pathway hierarchies and network analysis can also reduce the long list of relevant pathways resulting from a pathway analysis. However, once the relevant processes are identified, researchers still must investigate those pathways one by one, in order to understand in detail how a SNP influences gene function in the entire process. MetaCore and IPA are examples that use networks to visualize the data integration. However, genetic variants cannot be used readily with these methods, because the data uploaded are not completely recognized. Adding the variants option to these tools would allow the user to contextualize the function of the genetic polymorphisms on different molecular levels. In addition, when data such as SNP–SNP interactions become available, pathway tools that present a network visualization option (i.e., MetaCore and IPA) could support display of epistatic interactions from a set of SNPs located in genes that function in the same pathway. In general, several specific omics data integration methods that support inclusion of genetic variants in a pathway already exist. In this context, it is suitable to mention BioXM from Biomax Informatics (Maier et al., 2011) because it semantically integrates existing knowledge such as genotype–phenotype relations or signal transduction pathways, and organizes data into structured networks that are connected with clinical and experimental data (e.g., metabolites or proteomics datasets). With regard to the pathway collection, BioXM is flexible in that, it can display any pathway data, but requires input of pathway enrichment statistics from other sources. BioXM, on the other hand, is designed for flexibility and can integrate and display a wide range of relationships between entities, including pathways and genetic variants, but linking those two has not been demonstrated with GWAS data.

### New Types of Genetic Variant Interactions for Pathway-Based Analysis Tools

Additional characteristics regarding genetic variant interactions currently are rarely depicted in pathway visualizations: edgetics, gene–environment (G × E), and epistatic interactions. Edgetics is a new term referring to network perturbation models focusing on specific alterations of the molecular interactions resulting from genetic variants (Zhong et al., 2009). This perturbation model might improve understanding of how mutations associating with complex diseases affect biological networks or interactome properties (Markowetz, 2010). With network visualization already developed in some of the presented tools, it would be exciting to see this model implemented as a new feature.

Another area in which pathway visualization of genetic associations can be improved involves G × E, where the genotype–phenotype association exists only under certain environmental conditions. A recently published catalog of G × Es for numerous cardiometabolic phenotypes showed the wide extent under which the genotype–phenotype association can be modified by factors such as diet, exercise, sleep, and many other exposures and lifestyle factors (Parnell et al., 2014). For identical traits, that study noted sparse overlap of SNPs contributing to main-effect associations from GWAS compared to those supporting G × E interactions. In such instances, the pathway edges linking the G × E gene to the phenotype obviously would be conditional, and in many examples would contain entities such as glucose, palmitic acid, or linoleic acid, which are constituents of standard metabolic pathways. Finally, epistatic interactions were used here as a use case to test the visualization tool. As a result PathVisio, MetaCore, and IPA are the tools that support upload of variant data, and highlight those variants in the pathways of the genes related to the uploaded SNPs. This feature aids investigation of the effect of the epistatic SNPs within the genes and their pathways. However, only PathVisio is able to provide the complete list of variants present in the uploaded data. Indeed, IPA identifies the genes related to the SNPs without showing the SNPs, and MetaCore performed a SNP-gene mapping that resulted in a selection of genes not included in the original dataset. Concerning IPA, it is notable to mention that Ingenuity developed another software specifically dedicated to variant investigation called “Variant Analysis” that was not detected by the review literature search, but discovered only through the Ingenuity website. In addition, the PathVisio RegInt plugin, even if it can upload the complete dataset, fails to automatically provide to the users the overview of the total pathways that present at least one of the genes with the SNPs. This feature is supported by IPA and MetaCore. The epistatic obesity use case shows that IPA, MetaCore and PathVisio have several features that permit the visualization of genetic variants in pathways. However, these features are not harmonized in one tool, but this is a reasonable outcome because the tools were not built with the aim to analyze genetic variants. On the other hand, it is remarkable to notice that these tools already have some characteristics that, with improvements, could permit such complexities of variant analysis. In summary, such conditional relationships as epistasis, G × E interactions and edgetics will need to be considered for pathway-based visualization of association data because genome-wide approaches to identify such genetic elements are rapidly maturing (Markowetz, 2010; Parnell et al., 2014; Wei et al., 2014; De et al., 2015).

## Conclusion

What is especially needed regarding the SNP data visualization in pathway-based analysis tools are two important items. One, there must be development and integration in the tools of specific statistical methods for GWAS pathway analysis (red highlight in **Figure 1**). Two, improving strategies for combined visualization of genetic data with other omics data in a pathways context will vastly facilitate interpretation of results. For the first point, as indicated in Section “Results” and listed in Supplementary Table 1, some accepted statistical methods used for pathway analysis of GWAS data have been described. Our recommendation is to include at least one of these algorithms in pathway-based analysis tools that focus on GWAS data. This will enhance pathway-based analyses by increasing accuracy to detect significant pathways because of the specificity of the statistics for GWAS data. Additionally, it is necessary that results such as subpaths of genes with consideration of significant SNPs in the affected pathways, are visualized properly. Next, the necessity to identify a strategy of combining genetic variants with other omics data could be addressed by permitting immediate evaluation of significant SNPs in the pathway context. While a detailed report of functional information is already provided for genes in a pathway, this needs to be extended to SNPs. Examples of SNP information that could be useful to add include: (i) incorporation of data or links to databases that contain association data from other sources, including data mined from GWAS databases, epistasis and G × E, eQTL data, and allele-specific drug and micronutrient responses; (ii) SNP function and description; (iii) LD plot images anchored to the chromosomal region where the SNP maps. Lastly, other improvements in visualizing genotype–phenotype associations will involve extending the phenotype information to co-morbidities, and data from electronic health records and public health agencies.

The main aim of this review is to give an overview of the current state of the tools that visualize SNP data in a pathway context. We attempted to identify and describe the visualization options of the tools that resulted from a literature review in order to provide suggestions for improvements in this developing field (**Figure 1**). We also have reported the necessity to include in these tools statistical methods for the pathway-based analysis in GWAS, aiming to define features for more comprehensive pathway-based analysis tools.

## Author Contributions

EC performed the analysis and wrote the paper. LP and CE revised critically the work, and provided final approval with agreements on the content.

## Conflict of Interest Statement

EC, LP, and CE state that there are no conflicts of interest and there is no goal to endorse a commercial entity. EC and CE are part of the team that developed PathVisio, one of the visualization tools evaluated in this review.

## Footnotes

1 http://www.qiagen.com/ingenuity

2 https://portal.genego.com/

3 http://genapha.icapture.ubc.ca/PathTutorial/

4 http://www.caleydo.org/

5 http://www.genome.jp/kegg

6 http://www.wikipathways.org/

7 https://www.pathvisio.org/plugin/biomartconnect/

8 https://www.pathvisio.org/plugin/regint-plugin/

9 http://www.ensembl.org/biomart/martview

10 https://github.com/PathVisio/RegInt-Plugin/wiki/User-Guide

11 http://www.cancergenome.nih.gov/

12 http://genapha.icapture.ubc.ca/Path2Tutorial/
