# Supplementary material for: A Review of Pathway-Based Analysis Tools That Visualize Genetic Variants
Source: Front Genet. 2017 Nov 7;8:174. doi: 10.3389/fgene.2017.00174 (PMC5681904; doi:10.3389/fgene.2017.00174)
Supplement: Supplementary file 1 [file Data_Sheet_1.docx]

Supplementary Material

# A review of pathway-based analysis tools that visualise genetic variants

**Elisa Cirillo^1*^, Laurence D Parnell^2^, and Chris T Evelo^1^**

^1^ Department of Bioinformatics – BiGCaT, Maastricht University, Maastricht, The Netherlands.

^2^ Agricultural Research Service, USDA, Jean Mayer-USDA Human Nutrition Research Center on Aging at Tufts University, Boston, MA, USA

*** Correspondence:**Elisa Cirillo

elisa.cirillo@maastrichtuniversity.nl

# 1 Supplementary Table

**Table 1**: Studies merging interpretation of genetic variation data with pathway-based analysis

| **Publication** | **Data** | **Variants type** | **Bioinformatic Tool** | **Algorithm for Pathway analysis** |
| --- | --- | --- | --- | --- |
| Ballard, 2010 | GWAS | SNPs | - | Random set and Binomial approximation |
| Ballard, 2009 | GWAS | SNPs | - | Random set and Binomial approximation |
| Baranzini, 2009 | GWAS | SNPs | - | jActive modules |
| Beyene, 2009 | GWAS | SNPs | - | GSEA |
| Chang, 2008 | GWAS | SNPs | - | Random forest |
| Chen D, 2014 | GWAS | SNPs | - | SRT |
| Chen X, 2010 | GWAS | SNPs | - | SPCA |
| Chen Y, 2010 | GWAS | SNPs | - | GSEA |
| Chuang, 2013 | GWAS+Epigenetic | SNPs | - | GSEA, SUM, SQUARE STAT, SUM-STAT |
| Daneshjou, 2013 | GWAS | SNPs | - | Own approach |
| De las Fuentes, 2012 | GWAS | SNPs | - | VSEA |

| **Publication** | **Data** | **Variants type** | **Bioinformatic Tool** | **Algorithm for Pathway analysis** |
| --- | --- | --- | --- | --- |
| Donato, 2013 | GWAS | SNPs | - | Own approach |
| Eleftherohorinou, 2009 | GWAS | SNPs | - | Cumulative trend test statistic |
| Evangelou, 2012 | GWAS | SNPs | **-** | comparison of methods |
| Evangelou, 2014 | GWAS | SNPs | - | Bayesian hierarchical framework |
| Fehringer, 2012 | GWAS | SNPs | - | comparison of methods |
| Ghosh, 2013 | GWAS | SNPs, indels | - | Over Representation analysis (ORA ingenuity) and GSEA |
| Gu 2011 | somatic mutation | SNPs | - | Benjamini-Hochberg, 1995 and Hypergeometric distribution |
| Gui, 2011 | GWAS | SNPs | - | comparison of methods |
| Harari, 2012 | GWAS | SNPs | - | ALIGATOR |
| Helleman, 2010 | GWAS | SNPs | Ingenuity | IPA algorithm (software) |
| Holden, 2008 | GWAS | SNPs | - | GSEA-SNP |
| Hu, 2011 | GWAS | SNPs | - | Linear and logistic regression to assess variants in pathway |
| Inada, 2008 | GWAS | SNPs | Ingenuity | IPA algorithm (software) |
| Jia, 2011 | GWAS | SNPs | - | GSEA |
| Jia, 2012 | GWAS+expression | SNPs | - | comparison of methods |
| **Publication** | **Data** | **Variants type** | **Bioinformatic Tool** | **Algorithm for Pathway analysis** |
| Kar, 2013 | GWAS | SNPs | - | SRT |
| Lee Y.H., 2012 | GWAS | SNPs | - | ICSNPathway |
| Lee Y.H., 2012 | GWAS | SNPs | - | ICSNPathway |
| Lee Y.H., 2012 | GWAS | SNPs | - | ICSNPathway |
| Lee Y.H., 2013 | GWAS | SNPs | - | ICSNPathway |
| Lee Y.H., 2014 | GWAS | SNPs | - | ICSNPathway with I-GSEA algorithm |
| Lee Y.H., 2014 | GWAS | SNV, CNV | - | ICSNPathway I-GSEA algorithm |
| Lee D., 2013 | GWAS | SNPs | - | GSEA and ARTP |
| Leiserson, 2013 | somatic mutation | SNV, CNV, indel | - | Multi-Dendrix in a pipeline |
| Li D., 2012 | GWAS | SNP | - | ARTP |
| Luo, 2010 | GWAS | SNP | - | Linear combination test, quadratic test, decorrelation test |
| Martin 2010 | GWAS | SNP | - | Prioritizer (software) |
| Menashe, 2010 | GWAS | SNPs | - | Kolmogorov-Smirnov for enrichment score, and FDR |
| Menashe, 2012 | GWAS | SNPs | - | GSEA and ARTP |
| Mukherjee, 2014 | GWAS | SNPs | - | Sequential Kernel Association Test (for rare variants) |
| **Publication** | **Data** | **Variants type** | **Bioinformatic Tool** | **Algorithm for Pathway analysis** |
| Ngwa, 2011 | GWAS | SNP | Ingenuity | IPA statistics, GSEA and empirical enrichment p-value |
| O'Dushlaine, 2009 | GWAS | SNPs | - | SRT |
| Peng, 2010 | GWAS | SNPs | - | Statistics methods (Fisher, Sidak, Simes comb test, Hypergeom.) |
| Schoof, 2011 | GWAS | SNPs | - | Gene -set approach with SUMSTAT and SUMQ |
| Shahbaba, 2012 | GWAS | SNPs | - | Hierarchical Bayesian model, GSEA, ALIGATOR |
| Song, Lee, 2013 | GWAS | SNPs | MetaCore | Analyze Single Experiment workflow |
| Song, Choi, 2013 | GWAS | SNPs | - | ICSNPathway |
| Song, Lee, 2013 | GWAS | SNPs | - | ICSNPathway |
| Sun,2014 | GWAS | SNPs | - | iPEAP |
| Uzun, 2013 | GWAS | SNPs | - | GSEA |
| Verschuren, 2013 | GWAS | SNPs | - | PLINK, GRASS, ALIGATOR |
| Wang K, 2007 | GWAS+expression | SNPs | - | GSEA modified, (Subramanian, 2005) |
| Wang L., 2011 | GWAS | SNPs | - | Threshold-free, hierarchical generalized linear mixed |
| Weng, 2011 | GWAS | SNPs | - | SSEA (new approach) |
| Xie, 2014 | GWAS | SNPs | - | Gene set analysis toolkit V2 |
| **Publication** | **Data** | **Variants type** | **Bioinformatic Tool** | **Algorithm for Pathway analysis** |
| Yu, 2009 | GWAS | SNPs | - | ARTP |
| Zamar, 2009 | GWAS | SNPs | Path | Path statistic (software) |
| Zhang L., 2010 | GWAS | SNPs | - | Kolmogorov-Smirnov, sum-statistic |
| Zhang M., 2011 | GWAS | SNPs | - | Enrichment score, with Kolmogorov-Smirnov |
| Zhang M., 2012 | GWAS+expression | SNPs | - | Zhong methods (2010b), Kolmogorov-Smirnov |
| Zhang R., 2013 | GWAS | SNPs | - | GSEA |
| Zhao, 2011 | GWAS | SNPs | - | FPC- based test statistics |
| Zhao, 2014 | NGS | SNPs | **-** | SFPCA-based statistic approach |
| Zhong, 2010 | GWAS+expression | SNPs | - | GSEA |

ARTP: Adaptive rank truncated product, FDR: False Discovery Range, FPC: First Principal Component, GSEA: Gene Set Enrichment Analysis, ICSNPathway: Identify candidate casual SNPs and pathway, indels: insertion-deletion variants, LP: Limiting Pathway Model, SFPCA: Smoothed functional component analysis, SPCA: Principal Component Analysis, SQUARE STAT: Square statistics, SRT: SNP Ratio Test method, SSEA: SNP set enrichment analysis, SUM: Summary, SUM-STAT: Summary statistic, VSEA: Variable Set Enrichment Anaysis.

**Table 2**: Pathway analysis tools found in the second literature search (August 2016 - January 2017), which do not include interactive pathway visualisation features.

| **Publication** | **Data** | **Variants type** | **Bioinformatic Tool** | **Features** |
| --- | --- | --- | --- | --- |
| Kutmon, 2015 | Gene expression, GWAS | SNP | PathVisio3 | Pathway analysis and data visualisation on the pathway diagram |
| Lips, 2015 | GWAS | SNP | JAG | Self-contained and/or competitive tests for gene-set analysis |
| Wang, 2015 | GWAS | SNP | iPINBPA | An integrative protein interaction network based pathway analysis for sub-networks identification |
| Wen, 2016 | GWAS | SNP | PAPA | Enrichment analysis for the identification of pleiotropic pathways |

JAG: Joint Association of Genetic variant; iPINBPA: integrative protein-interaction-network-based pathway analysis.

# 2 Supplementary Figures


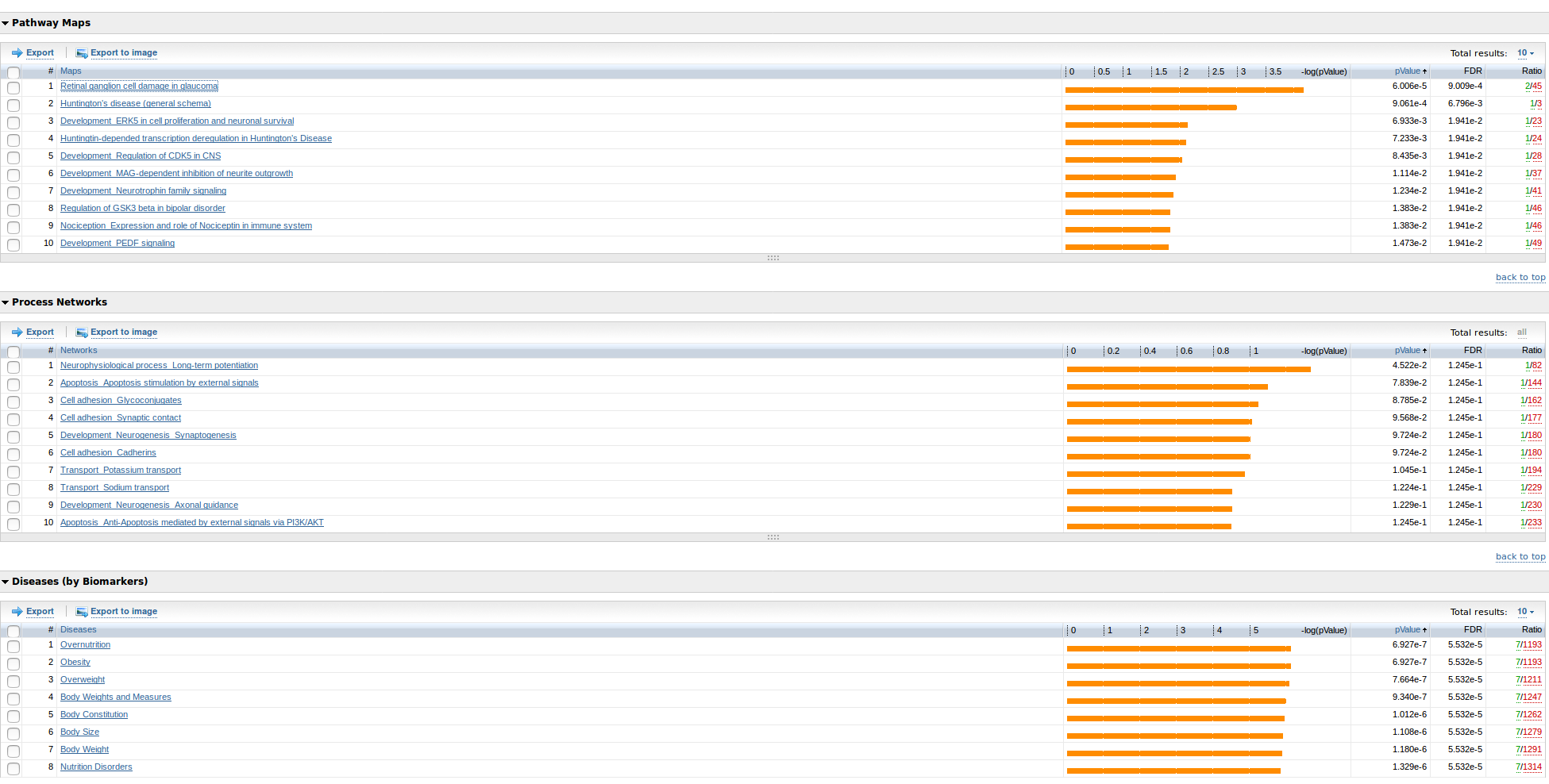


**Supplementary Figure 1.** Overview of the Enrichment Analysis Workflow results from MetaCore. Different outputs such as pathway maps, process networks, diseases (symbolized by biomarkers) and gene ontology (GO) processes (not shown in the figure) are listed as clickable items (blue text on the left side of the image). Each item is ordered by: enrichment p-value indicated by orange bars and values, false discovery rate (FDR) corrections, and ratio of the genes identified in the biological process (on the right side of the image).


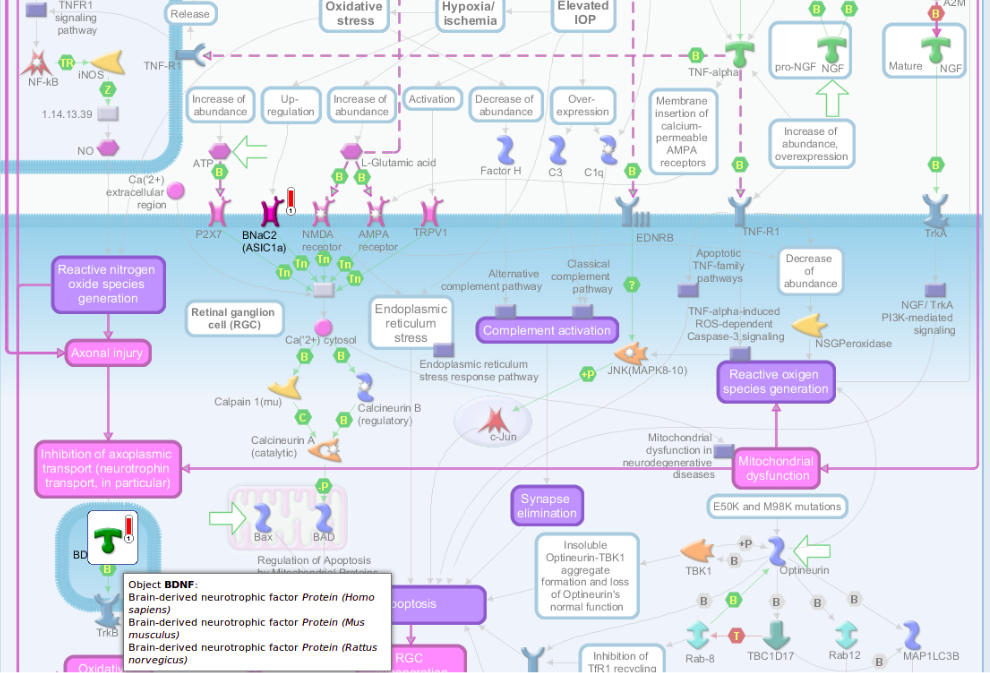


**Supplementary Figure 2.** MetaCore pathway of “Retinal ganglion cell damage in glaucoma”. The two genes in the pathway – *BDNF* and *BnaC2 (ASIC1a)*– that contain input SNPs from the dataset appear as more brightly colored elements, each with a red bar. Clicking a gene symbol displays the HGCN gene name and the available species information. In this example detailed information on the description of the gene and encoded protein for human, mouse and rat are accessible in another window (not shown). Clicking the red bar gives details on the uploaded data of that gene, in this case the SNP rsID (not shown).


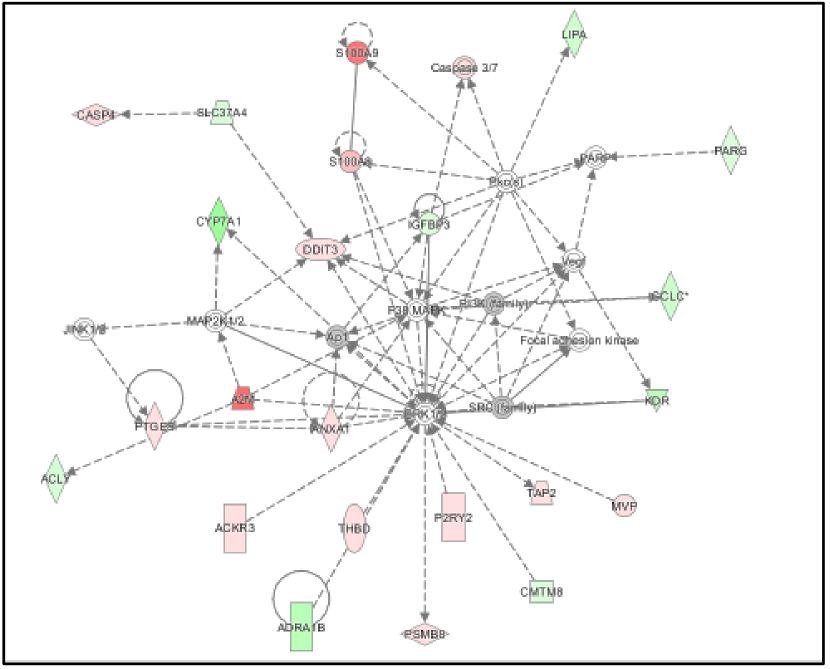


**Supplementary Figure 3.** IPA gene network, in which genes are represented as nodes with different colors and shapes and the gene connections are displayed with edges between nodes. The gene nodes are clickable and they link to a page with additional information related to that gene, including biochemical elements, metabolites, and references curated by the IPA team (not shown). No hyperlinks, information or symbols about the uploaded SNPs are able to be displayed in the network figure.


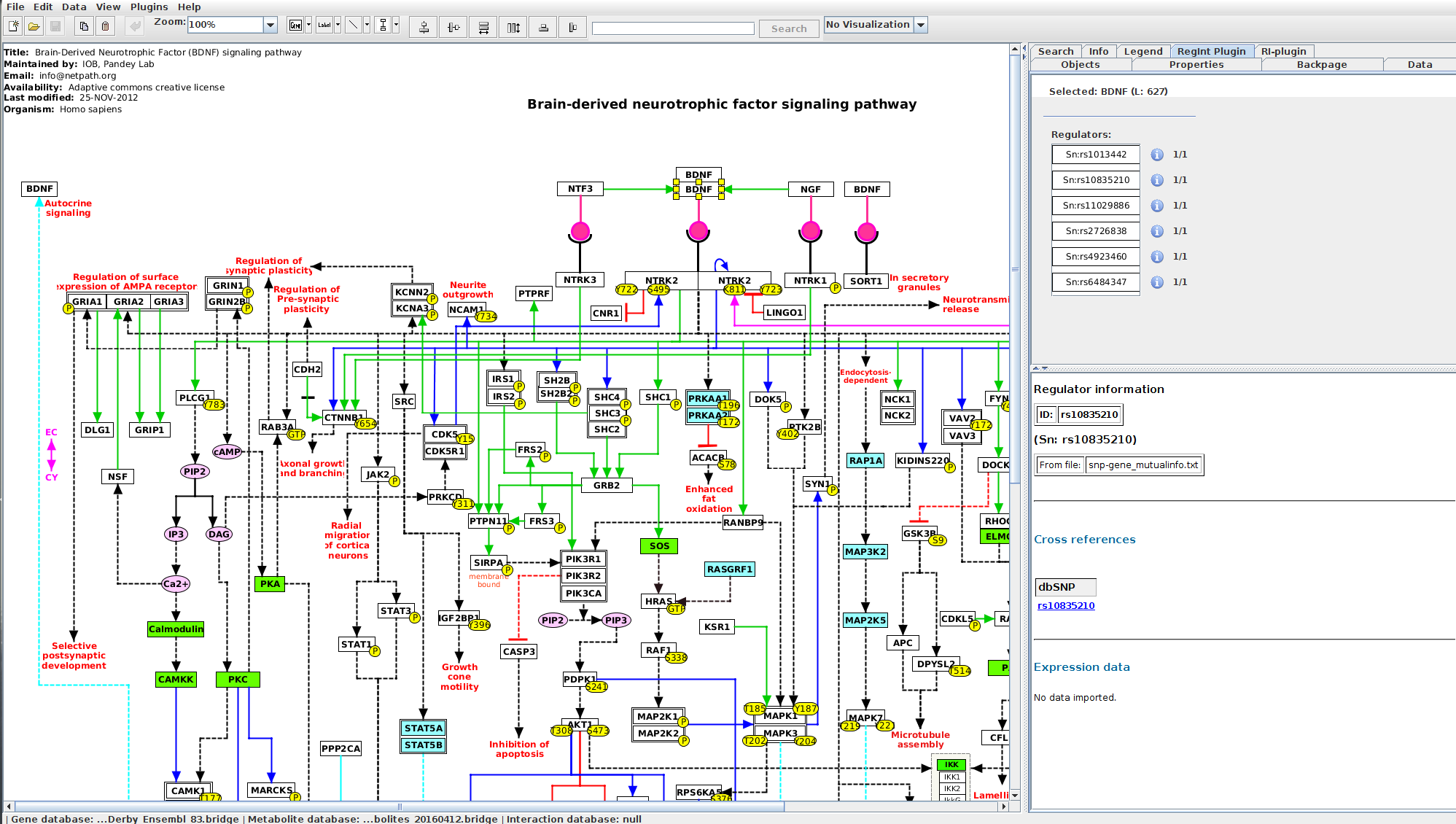


**Supplementary Figure 4.** PathVisio view of the “brain-derived neurotrophic factor signaling pathway”. At the top of the figure the *BDNF* gene is clicked (the element highlighted with yellow boxes). In the backpage panel, on the right, different types of information related to the SNP uploaded for that gene are shown. The SNP rsIDs located in the gene are listed under the **Regulators** section in the upper side. When one of the blue circles right of the SNP ID is selected, the SNP hyperlink is shown in dark blue, in the **Cross references** section. The hyperlink connects to the dbSNP database in which the SNP description is provided (not shown). Additional information such as **Expression data** can be visualised in the bottom right section.


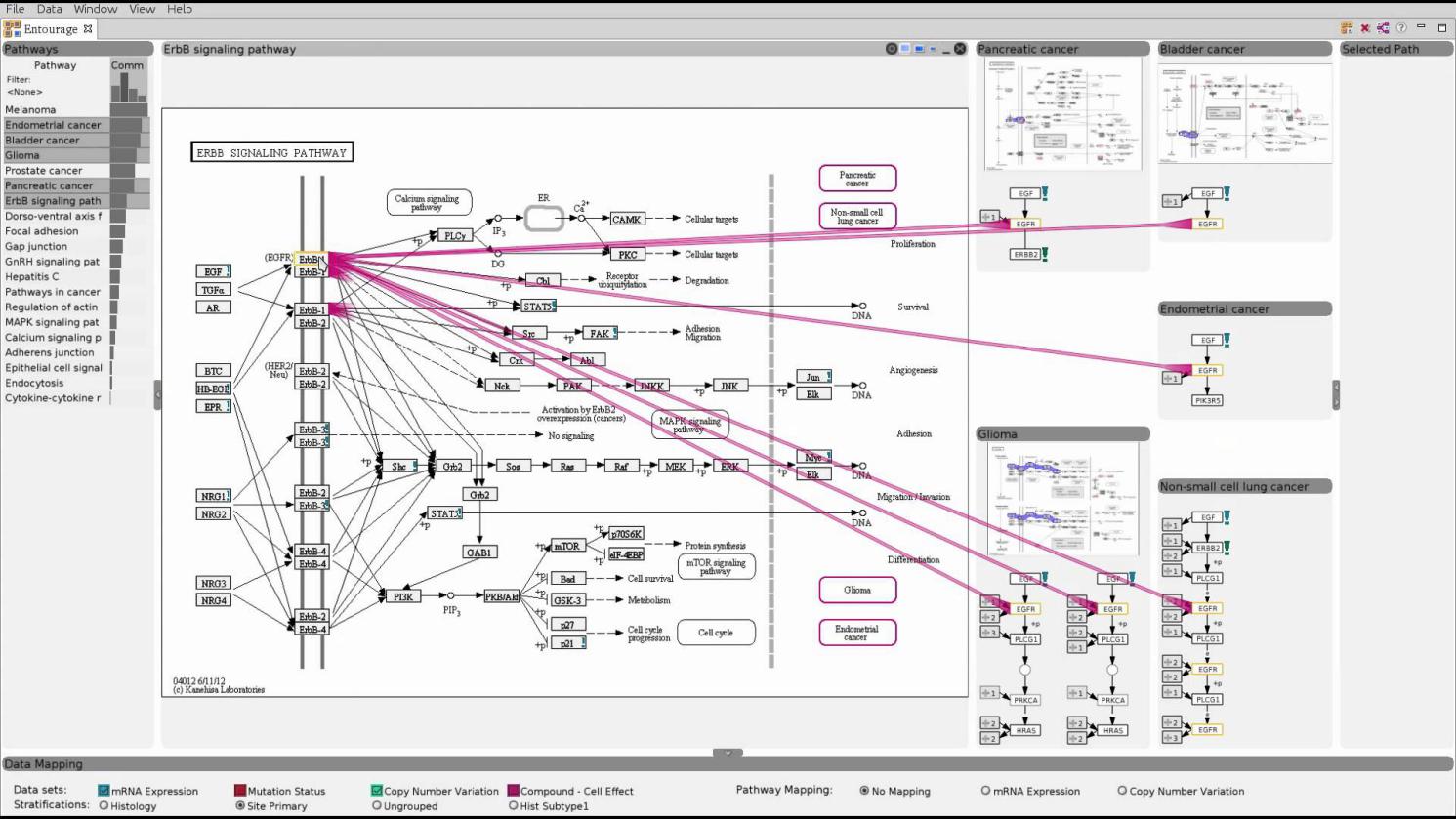


**Supplementary Figure 5.** The Entourage view from Calyedo shows different pathways connected with purple lines because of the presence of the same gene in each pathway map. The *EGFR* gene is selected in the “EGFR signaling pathway”, which then prompts the display of purple lines that connect the selected genes from the main pathway to their occurrence in other pathways or sub-pathways, as displayed in the right side of the figure.
